# CALGB 80802 (Alliance): Impact of Sorafenib with and without Doxorubicin on Hepatitis C Infection in Patients with Advanced Hepatocellular Carcinoma

**DOI:** 10.1158/2767-9764.CRC-22-0516

**Published:** 2024-03-07

**Authors:** Ghassan K. Abou-Alfa, Susan M. Geyer, Andrew B. Nixon, Federico Innocenti, Qian Shi, Priya Kumthekar, Sawyer Jacobson, Imane El Dika, Amin Yaqubie, Juan Lopez, Binhui Huang, Yi-Wei Tang, Yujia Wen, Lawrence H. Schwartz, Anthony B. El-Khoueiry, Jennifer J. Knox, Lakshmi Rajdev, Monica M. Bertagnolli, Jeffrey A. Meyerhardt, Eileen M. O'Reilly, Alan P. Venook

**Affiliations:** 1 Memorial Sloan Kettering Cancer Center, New York, New York.; 2 Weill Medical College of Cornell University, New York, New York.; 3 Alliance Statistics and Data Management Center, Mayo Clinic, Rochester, Minnesota.; 4 Duke Cancer Institute, Duke University Health System, Durham, North Carolina.; 5 University of North Carolina, Chapel Hill, North Carolina.; 6 Alliance for Clinical Trials in Oncology Protocol Office, Chicago, Illinois.; 7 University of Chicago, Chicago, Illinois.; 8 Columbia University Medical Center, New York, New York.; 9 New York-Presbyterian Hospital, New York, New York.; 10 USC Norris Comprehensive Cancer Center, Los Angeles, California.; 11 Princess Margaret Cancer Centre, Toronto, Ontario, Canada.; 12 Lenox Hill Hospital, New York, New York.; 13 Brigham and Women's Hospital, Boston, Massachusetts.; 14 Dana-Farber/Partners Cancer Care, Boston, Massachusetts.; 15 UCSF Helen Diller Family Comprehensive Cancer Center, San Francisco, California.

## Abstract

Sorafenib blocks nonstructural protein 5A (NS5A)-recruited c-Raf–mediated hepatitis C virus (HCV) replication and gene expression. Release of Raf-1-Ask-1 dimer and inhibition of Raf-1 via sorafenib putatively differ in the presence or absence of doxorubicin. Cancer and Leukemia Group B (CALGB) 80802 (Alliance) randomized phase III trial of doxorubicin plus sorafenib versus sorafenib in patients with advanced hepatocellular carcinoma (HCC), showed no improvement in median overall survival (OS). Whether HCV viral load impacts therapy and whether any correlation between HCV titers and outcome based on HCV was studied. In patients with HCV, HCV titer levels were evaluated at baseline and at multiple postbaseline timepoints until disease progression or treatment discontinuation. HCV titer levels were evaluated in relation to OS and progression-free survival (PFS). Among 53 patients with baseline HCV data, 12 patients had undetectable HCV (HCV-UN). Postbaseline HCV titer levels did not significantly differ between treatment arms. One patient in each arm went from detectable to HCV-UN with greater than 2 log-fold titer levels reduction. Aside from these 2 HCV-UN patients, HCV titers remained stable on treatment. Patients who had HCV-UN at baseline were 3.5 times more likely to progress and/or die from HCC compared with HCV detectable (HR = 3.51; 95% confidence interval: 1.58–7.78; *P* = 0.002). HCV titer levels remained unchanged, negating any sorafenib impact onto HCV titer levels. Although an overall negative phase III study, patients treated with doxorubicin plus sorafenib and sorafenib only, on CALGB 80802 had worse PFS if HCV-UN. Higher levels of HCV titers at baseline were associated with significantly improved PFS.

**Significance:**

Sorafenib therapy for HCC may impact HCV replication and viral gene expression. In HCV-positive patients accrued to CLAGB 80802 phase III study evaluating the addition of doxorubicin to sorafenib, HCV titer levels were evaluated at baseline and different timepoints. Sorafenib did not impact HCV titer levels. Despite an improved PFS in patients with detectable higher level HCV titers at baseline, no difference in OS was noted.

## Introduction

Sorafenib efficiently blocks nonstructural protein 5A (NS5A)-recruited c-Raf–mediated hepatitis C virus (HCV) replication and viral gene expression (1). Sorafenib a highly specific c-Raf inhibitor and mediated antiviral effects were demonstrated to negate HCV replication and viral gene expression (2). In HCV-replicating cells, sorafenib also decreased the hyperphosphorylated form of NS5A that led to the formation of added hypophosphorylated forms. In comparison, inhibition of targets downstream of c-Raf or inhibition of other tyrosine kinase inhibitors like sunitinib did not affect HCV replication. HCV infection of Huh-7 cells has been shown to induce VEGF expression that depolarizes hepatocytes and disrupts tight junctions, thereby making cells more susceptible to further HCV infection in an autocrine fashion (3). Sorafenib treatment inhibited this cycle of events. These data highlight a potential role for VEGF antagonists to treat HCV infection in addition to their intrinsic properties of regulating hepatocellular carcinoma (HCC) growth and development.

Release of Raf-1-Ask-1 dimer and inhibition of Raf-1 via sorafenib putatively differ in the presence or absence of doxorubicin. Doxorubicin-induced cytotoxicity is mediated by the proapoptotic ASK-1 pathway. Basic FGF activity has been associated with expression of Raf-1, which binds to and neutralizes ASK1, preventing the cell from undergoing apoptosis. Inhibition of Raf-1 by sorafenib would be expected to restore chemosensitivity to doxorubicin (4). Potential effects of Raf onto the sensitivity of cells to doxorubicin and in regulation of the multidrug resistance-1 (mdr-1) gene led to the consideration of overcoming doxorubicin resistance by combining it with anti-Raf (5, 6). This led to a phase I study evaluating the addition of doxorubicin to sorafenib. Four patients with HCC achieved prolonged stability of disease and continued therapy for more than 1 year. Despite a 21% AUC increase of doxorubicin when administered concomitantly with sorafenib, no substantial worsening of toxicity over what would be expected from either compound administered individually (7). Collectively, the above led to a positive randomized phase II clinical trial of doxorubicin plus sorafenib versus doxorubicin (8). The finding of greater median time to progression, overall survival (OS), and progression-free survival (PFS) with sorafenib plus doxorubicin compared with doxorubicin monotherapy led to Cancer and Leukemia Group B (CALGB) 80802, a randomized phase III trial that evaluated doxorubicin and sorafenib combination versus sorafenib in patients with advanced HCC (9). CALGB is now part of the Alliance for Clinical Trials in Oncology (Alliance). In addition, an analysis of prognostic factors of another two randomized phase III studies of sorafenib alone suggested better survival in patients with HCV albeit no correlative analyses were performed (10). Patients were accrued from 2010 to 2015, which predated the availability of anti-HCV therapies.

Despite the pretrial hypothesis of a favorable drug–drug interaction, CALGB 80802 did not show an improvement in median OS with the addition of doxorubicin to sorafenib compared with sorafenib alone. Median OS was 9.3 months [95% confidence interval (CI): 7.3–10.8 months] in the doxorubicin plus sorafenib arm and 9.4 months (95% CI: 7.3–12.9 months) in the sorafenib alone arm (HR, 1.05; 95% CI: 0.83–1.31). Anticipating the importance of HCV in the outcome of patients with advanced HCC, preplanned study objectives included an analysis of the possible antiviral effect of therapy on HCV, perhaps above and beyond effects on the cancer itself. The main objective of the correlative study reported herein was to evaluate HCV titers and change with treatment and to examine the correlation with patient outcome.

## Materials and Methods

Study investigators obtained written informed consent from all subjects. The studies were conducted in accordance with the Declaration of Helsinki, and they were approved by the Institutional Review Board (IRB) at all sites and/or the NCI central IRB and was registered (ClinicalTrials.gov NCT01015833). This unblinded randomized phase III clinical trial was led by Alliance in collaboration with Canadian Cancer Trials Group, Eastern Cooperative Oncology Group-American College of Radiology Imaging Network, and Southwest Oncology Group. It was launched in February 2010 and completed in May 2015; data were also analyzed during this time frame. Patients with histologically proven advanced HCC, no prior systemic therapy, Child-Pugh grade A score, Eastern Cooperative Oncology Group performance status of 0 to 2 (later amended to 0–1), and adequate hematologic, hepatic, renal, and cardiac function were eligible. The OS primary endpoint had a final analysis planned with 364 events observed among 480 total patients with 90% power to detect a 37% increase in median OS.

To determine the endpoint of HCV on outcomes with sorafenib treatment in each arm, preplanned study objectives were used to determine the proportion of patients with undetectable viral load (<50 copies/mL), and to assess and compare the HCV antiviral effect of sorafenib or sorafenib plus doxorubicin. That was performed by comparing patients’ rate of undetectable viral load or 2-log decline in viral load, and association with OS, PFS, and response rate for the whole study population, and on each treatment arm. The rate of virologic failure, defined as reversing from undetectable (<50 copies per mL) to detectable viral load, or increase in 2 logs above nadir, at any point in time was also to be assessed.

Peripheral venous blood was collected at baseline and on-treatment from patients who provided written informed consent. Samples were processed on-site within 2 hours; serum was aliquoted, frozen, and shipped on dry ice to the Alliance Pathology Coordinating Office for centralized storage. Aliquots of frozen serum were shipped to the Memorial Sloan Kettering lab for all HCV analyses. HCV RNA levels were measured by the Roche cobas AmpliPrep/cobas TaqMan HCV Test as described previously (11). Specimens were evaluated by PCR and genotyped at baseline on day 1 of cycle 1, and HCV titer levels were evaluated on day 1 of cycles 2, 3, and 4 as well as at time of disease progression and end of active treatment. Detectable HCV titers were defined as HCV RNA ≥50 copies/mL. Quasispecies changes for resistant mutations were investigated for patients who exhibited “virologic failure” (a reversal from undetectable <50 copies/mL to detectable ≥50 copies/mL viral load or an increase of 2 logs above nadir) during the study. Samples were stored and analyzed at the end of the follow-up period. Laboratory investigators were blinded to patient identity and outcomes. Influence of the HCV titer levels was evaluated in relation to the study objectives OS and PFS.

### Biostatistics

The prevalence of HCV with or without presence of hepatitis B virus (HBV) was evaluated across all patients. Those with HCV at baseline who had stored serum had HCV RNA and titer levels assessed. Summary statistics were calculated across all patients with HCV at baseline. We further summarized and compared participants who had serum versus those who did not have serum available for these analyses.

The number of copies/mL within each treatment group and across all patients was summarized at each timepoint. Differences over time and between treatments were assessed graphically, and fold changes were calculated in relation to baseline. Viral loads were evaluated as a continuous measure as well as categorized as undetectable versus detectable (<50 copies/mL; ≥50 copies/mL). A subset of patients did not have titer data available at baseline; given the observed consistency of titer levels over time, we also evaluated the influence of HCV from the first sample, where we imputed missing baseline measures using cycle 2 day 1 measures. Analyses were conducted using only those with baseline samples and using all patients with baseline values imputed.

The influence of HCV titer levels was evaluated in relation to PFS and OS. PFS was defined as the time from randomization to the progression and/or death due to any cause, where patients who were progression-free at their last evaluation were censored at that time. OS was defined as the time from randomization to death due to any cause, where patients alive at last follow-up were censored at that time. Kaplan–Meier methods were used to evaluate differences among those with detectable versus HCV undetectable titer levels. Cox proportional hazards models were used to evaluate the influence of continuous HCV titers and detectable versus undetectable HCV status in relation to PFS and OS, stratifying by treatment arm and by exploring potential effect modification of treatment arm (sorafenib with vs. without doxorubicin).

Data collection and statistical analyses were conducted by the Alliance Statistics and Data Management Center. Data quality was ensured by review of data by the Alliance Statistics and Data Management Center and by the study chairperson following Alliance policies. All analyses were based on the clinical data used for the primary clinical article for this trial (6).

### Data Availability

Deidentified patient data may be requested from Alliance for Clinical Trials in Oncology via concepts@alliancenctn.org if data are not publicly available. A formal review process includes verifying the availability of data, conducting a review of any existing agreements that may have implications for the project, and ensuring that any transfer is in compliance with the IRB. The investigator will be required to sign a data release form prior to transfer.

## Results

Of 356 patients enrolled on CALGB 80802, 83 patients were HCV-positive at baseline. Of note, the HCV infection rate was significantly higher in Black/African American patients (25/50 = 50%) compared with White patients (54/239 = 23%) or other race groups (4/67 = 6%; *P* < 0.0001; Table 1).

**TABLE 1:** Patient characteristics by HCV titer status on first available sample, for patients with available sample

	HCV titer status on first sample		
Characteristic	Detectable *N* = 41	Undetectable *N* = 12	All patients *N* = 53
*Age* *(years)*			
Median	61	63	61
Mean	61.122	59.667	60.792
SD	6.965	12.514	8.416
Range	51.000–85.000	30.000–72.000	30.000–85.000
*P*-value	0.603		
*Arm*			
Arm A: Doxorubicin + Sorafenib	19 (46.3%)	7 (58.3%)	26 (49.1%)
Arm B: Sorafenib	22 (53.7%)	5 (41.7%)	27 (50.9%)
*P*-value	0.465		
*ECOG PS*			
Median	1	1	1
Mean	0.683	0.833	0.717
SD	0.521	0.389	0.495
Range	0.000–2.000	0.000–1.000	0.000–2.000
*P*-value	0.36		
*Sex*			
Female	4 (9.8%)	0 (0.0%)	4 (7.5%)
Male	37 (90.2%)	12 (100.0%)	49 (92.5%)
*P*-value	0.26		
*Race*			
Black or African American	15 (36.6%)	0 (0.0%)	15 (28.3%)
White	25 (61.0%)	11 (91.7%)	36 (67.9%)
Not reported/Unknown/Missing	1 (2.4%)	1 (8.3%)	2 (3.8%)
*P*-value	0.038		
*Ethnicity*			
Hispanic or Latino	1 (2.4%)	2 (16.7%)	3 (5.7%)
Non-Hispanic	40 (97.6%)	10 (83.3%)	50 (94.3%)
*P*-value	0.061		
*Disease status*			
Locally advanced	23 (56.1%)	4 (33.3%)	27 (50.9%)
Metastatic	18 (43.9%)	8 (66.7%)	26 (49.1%)
*P*-value	0.165		
*Tumor grade*			
Moderately differentiated	17 (41.5%)	9 (75.0%)	26 (49.1%)
Poorly differentiated	8 (19.5%)	2 (16.7%)	10 (18.9%)
Well-differentiated	6 (14.6%)	1 (8.3%)	7 (13.2%)
Grade cannot be assessed	10 (24.4%)	0 (0.0%)	10 (18.9%)
*P*-value	0.146		
*T stage*			
T1	1 (2.5%)	0 (0.0%)	1 (1.9%)
T2	11 (27.5%)	1 (8.3%)	12 (23.1%)
T3	25 (62.5%)	8 (66.7%)	33 (63.5%)
T4	3 (7.5%)	3 (25.0%)	6 (11.5%)
Missing/not reported	1	0	1
*P*-value	0.236		
*N stage*			
N0	24 (60.0%)	6 (50.0%)	30 (57.7%)
N1	16 (40.0%)	6 (50.0%)	22 (42.3%)
Missing/not reported	1	0	1
*P*-value	0.539		
*M stage*			
M0	23 (56.1%)	4 (33.3%)	27 (50.9%)
M1	18 (43.9%)	8 (66.7%)	26 (49.1%)
*P*-value	0.165		
*ALT*			
Median	61	47.5	54
Mean	67.537	57.167	65.189
SD	35.329	37.177	35.661
Range	17.000–156.000	21.000–137.000	17.000–156.000
*P*-value	0.381		
*AST*			
Median	89	77.5	89
Mean	97.878	88.167	95.679
SD	53.773	57.223	54.165
Range	29.000–281.000	29.000–208.000	29.000–281.000
*P*-value	0.59		
*B* *ilirubin*			
Median	0.8	1	0.8
Mean	0.859	0.9	0.868
SD	0.345	0.372	0.348
Range	0.300–1.800	0.400–1.600	0.300–1.800
*P*-value	0.72		
*PT INR*			
Median	1.1	1.1	1.1
Mean	1.129	1.167	1.138
SD	0.228	0.115	0.208
Range	0.900–2.200	1.100–1.500	0.900–2.200
*P*-value	0.588		

Among the 83 HCV-positive patients at baseline, serum and thus HCV titer data were available on 53 patients. Per arm data are featured in Supplementary Fig. S1. Among these 83 HCV-positive patients, the only baseline clinical factor that was significantly different was that those in whom serum was available tended to have a higher percentage of Eastern Cooperative Oncology Group performance score (ECOG PS) >0 relative those who did not have serum available (68.6% vs. 41.3%, respectively; *P* = 0.042).

Of the 53 patients with HCV titer data, 12 patients (doxorubicin + sorafenib: 5, and sorafenib: 7) had undetectable HCV titer at their first measurement. The HCV titer levels did not significantly differ between treatment arms and the values remained very consistent over time (Supplementary Fig. S2). One patient in each arm went from detectable to HCV-UN and in so doing had a >2 log reduction in their titer levels from baseline. On treatment, 39 patients had HCV- detectable while 14 were HCV-UN (sorafenib + doxorubicin: 8, sorafenib: 6 patients).

**FIGURE 1:**
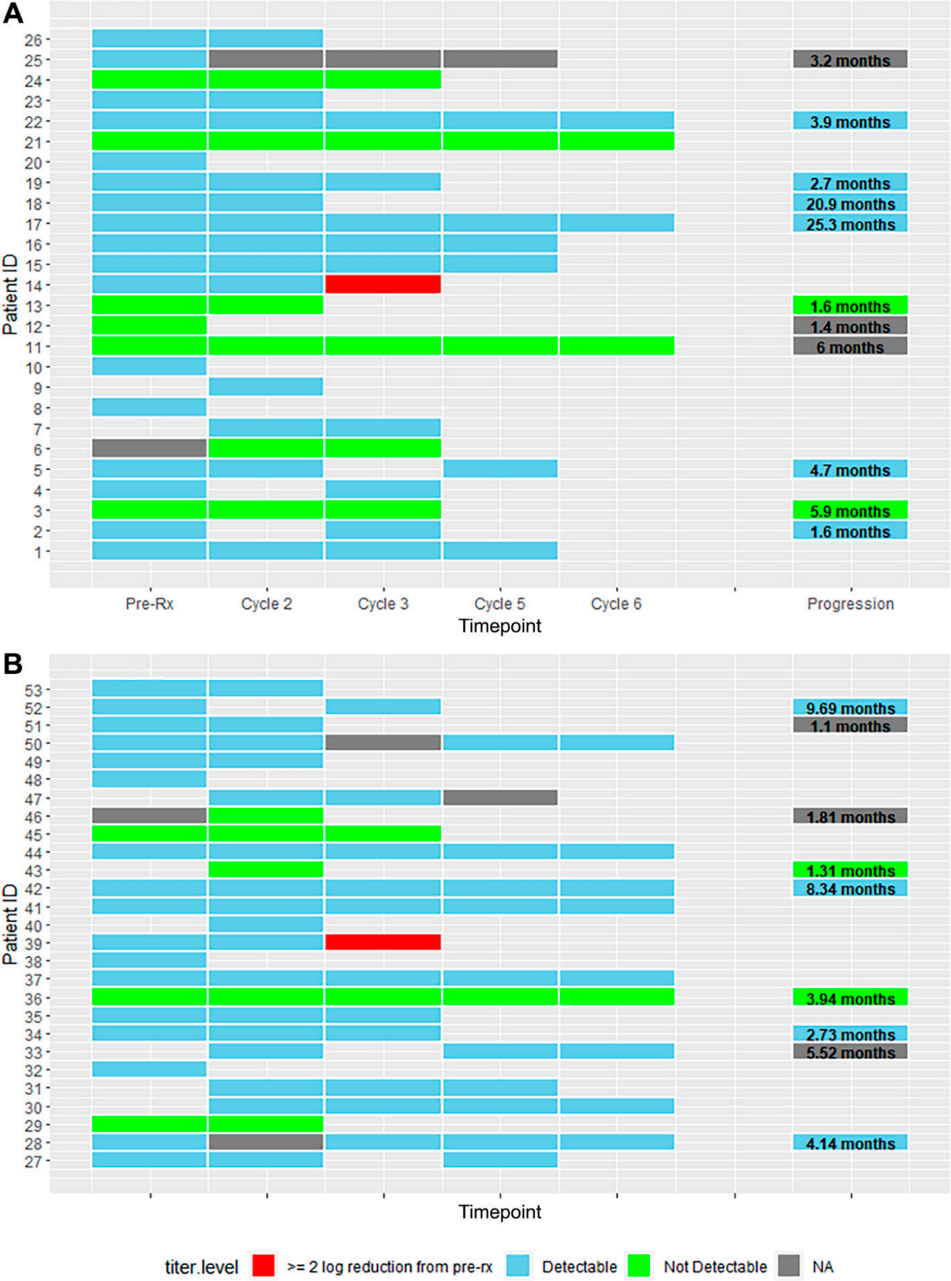
Swimmers plots of HCV viral titers classification (detectable, undetectable, ≥2 log reduction from baseline) over time for patients treated with doxorubicin and sorafenib (**A**) or sorafenib alone (**B**). For samples collected at the progression timepoint, the timing of progression is reflected as text in that tile.

Viral load over time per treatment arm is illustrated in Fig. 1A for doxorubicin plus sorafenib (A), and in Fig. 1B for Sorafenib alone. In view of the consistency of the titer levels, we imputed the cycle 2 day 1 HCV titer status (detectable vs. not) for patients who were missing baseline samples, albeit this presumption could not be validated. For the 2 patients who had a significant drop in HCV titer levels, this occurred by the cycle 3 day 1 timepoint. Among the 12 patients who had undetectable HCV at their first sample, 8 had measurements taken from pretreatment cycle 1 day 1 baseline sample and 4 were from a cycle 2 day 1 sample.

### Patient Outcomes

The study was halted after accrual of 356 patients with a futility boundary crossed at a planned interim analysis. In the overall trial, median OS was 9.3 months (95% CI: 7.3–10.8 months) in the doxorubicin plus sorafenib arm and 9.4 months (95% CI: 7.3–12.9 months) in the sorafenib alone arm (HR, 1.05; 95% CI: 0.83–1.31). The median PFS was 4.0 months (95% CI: 3.4–4.9 months) in the doxorubicin plus sorafenib arm and 3.7 months (95% CI: 2.9–4.5 months) in the sorafenib alone arm (HR, 0.93; 95% CI: 0.75–1.16).

**FIGURE 2:**
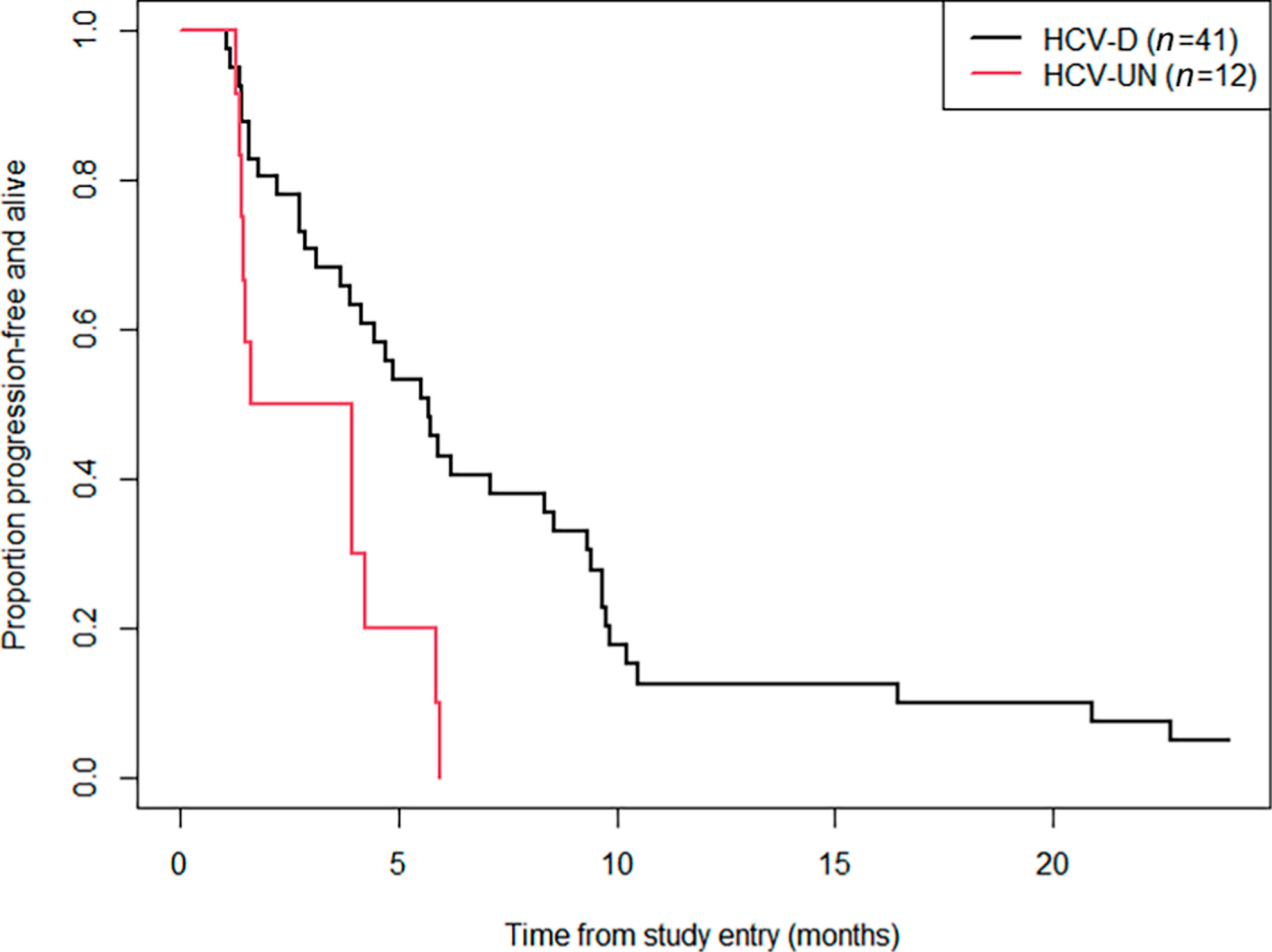
Kaplan–Meier curves for the PFS distributions for patients who had detectable (HCV-d) versus undetectable (HCV-UN) HCV titer levels on their first evaluated sample. The HR based on a corresponding Cox proportional hazards model was 3.51 for those with HCV-UN (vs. HCV-d), 95% CI: 1.58–7.78 (*P* = 0.002).

In this subset of 53 HCV-positive patients with HCV titer data available, the median follow-up was 36.1 months (95% CI: 34.7 to not reached) and 50 patients had a PFS event (progression and/or death) and 47 patients died. Median PFS for all patients across both treatment arms was 5.5 versus 2.8 months for HCV-detectable versus undetectable viral load, respectively (Fig. 2).

**FIGURE 3:**
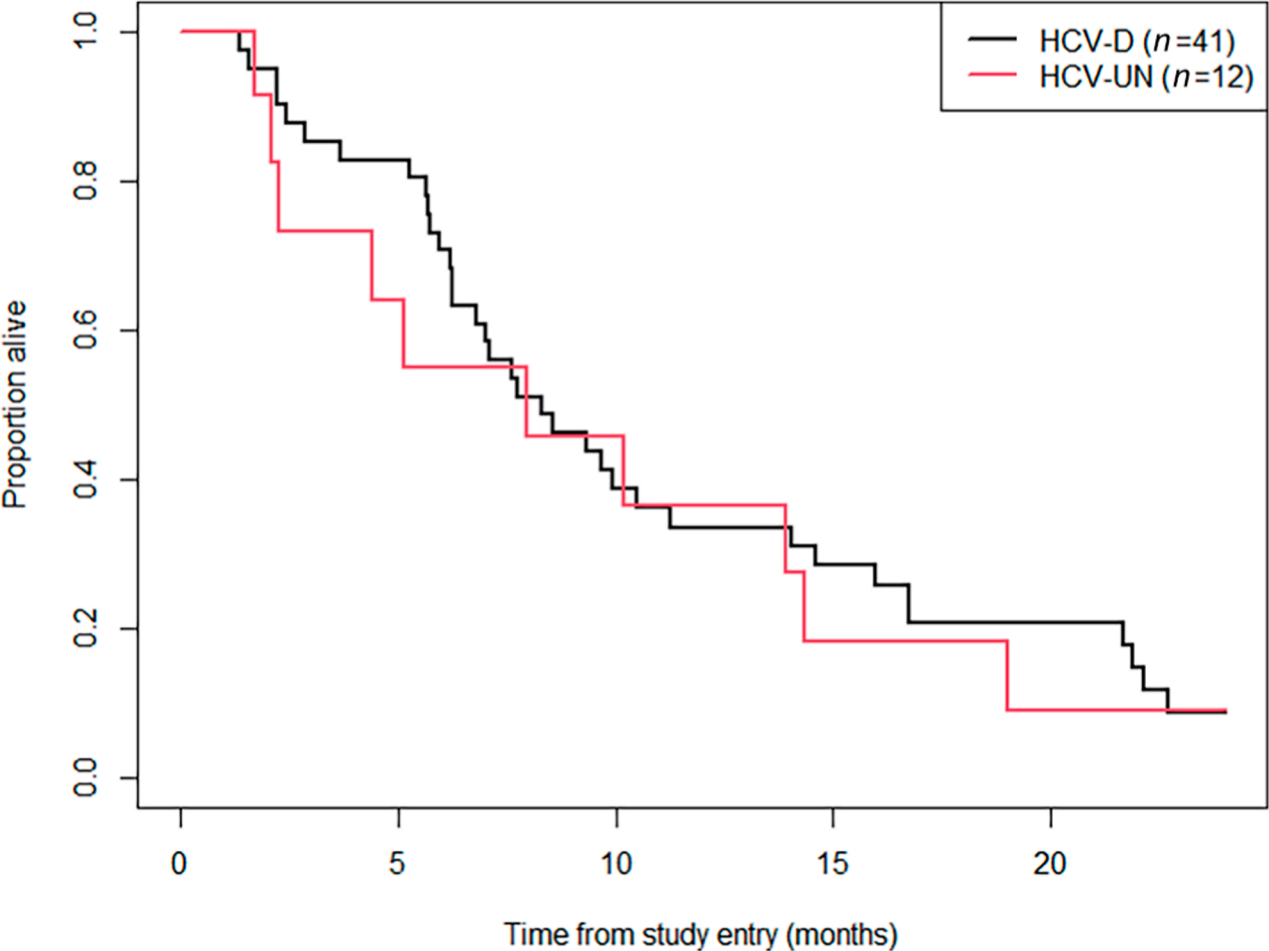
Kaplan–Meier curves for the OS distributions for patients who had detectable (HCV-d) versus undetectable (HCV-UN) HCV titer levels on their first evaluated sample. The HR based on a corresponding Cox proportional hazards model was 1.14 for those with HCV-UN (vs. HCV-d), 95% CI: 0.58–2.23 (*P* = 0.70).

PFS was significantly worse in those with undetectable HCV titer levels at their first sample timepoint compared with those with detectable HCV (HR = 3.51, 95% CI: 1.58–7.78; *P* = 0.002). This result held even when the analysis was restricted only to those individuals with baseline samples (HR = 3.47, 95% CI: 1.47–8.17; *P* = 0.004; Table 2). Differences in PFS and OS between HCV-detectable and HCV-UN viral load both at baseline and postbaseline are delineated in Table 2. These differences did not translate into significant differences in OS (HR = 1.14, 95% CI: 0.58–2.23; *P* = 0.70; Fig. 3). These results were consistent even when looking at results by treatment arm (Supplementary Figs. S3 and S4).

**TABLE 2:** Cox proportional hazards model results for PFS and OS. Each row reflects a model with that variable; models across all patients are stratified on treatment arm. Baseline HCV only includes those with pretreatment HCV titer data available (*n* = 43), and first sample HCV includes baseline HCV titer data for those with it available and cycle 2 data for those missing baseline (*n* = 53)

	*PFS*			*OS*		
	Median in months	HR (95% CI)	*P*-value	Median in months	HR (95% CI)	*P*-value
*All patients (N* *=* *53)*						
Baseline HCV-UN (vs. HCV-d)	1.6 (vs. 5.75)	3.47 (1.47–8.17)	0.004	5.2 (vs. 8.4)	1.57 (0.70–3.51)	0.28
First sample HCV-UN (vs. HCV-d)	2.8 (vs. 5.7)	3.51 (1.58–7.78)	0.002	7.95 (vs. 8.3)	1.19 (0.59–2.43)	0.63
Postbaseline HCV-UN (vs. HCV-d)	2.8 (vs. 5.7)	3.00 (1.48–6.12)	0.002	7.95 (vs. 8.3)	1.14 (0.58–2.23)	0.70
*D+S patients (N* *=* *26*)						
Baseline HCV-UN (vs. HCV-d)	2.8 (vs. 7.1)	3.55 (1.18–10.65)	0.024	6.55 (vs. 9.3)	1.37 (0.49–3.82)	0.55
First sample HCV-UN (vs. HCV-d)	3.9 (vs. 7.1)	3.26 (1.17–9.10)	0.024	7.95 (vs. 9.33)	1.36 (0.52–3.51)	0.53
Postbaseline HCV-UN (vs. HCV-d)	1.4 (vs. 3.9)	4.12 (1.47–11.52)	0.007	9.1 (vs. 8.95)	1.22 (0.49–3.03)	0.67
*S patients (N* *=* *27*)						
Baseline HCV-UN (vs. HCV-d)	1.5 (vs. 4.5)	3.35 (0.85–13.26)	0.085	2.3 (vs. 7.0)	1.98 (0.55–7.07)	0.30
First sample HCV-UN (vs. HCV-d)	1.5 (vs. 5.2)	3.91 (1.13–13.6)	0.032	8.3 (vs. 7.3)	1.02 (0.34–3.02)	0.97
Postbaseline HCV-UN (vs. HCV-d)	2.7 (vs. 4.9)	2.22 (0.78–6.28)	0.13	7.75 (vs. 7.0)	1.06 (0.39–2.86)	0.91

Abbreviation: HCV-d, HCV-detectable.

### Genotype

In addition to the HCV titer data, we also assessed the corresponding genotype data for these patients. Of the 53 patients with measurable samples, 12 were classified as “undetected” for the genotype. Of the remaining 41 patients, the majority (*n* = 23) were 1a, with 8 patients as 1b, and 2 additional patients as 1 who were not able to be further differentiated as 1a versus 1b. The remaining 7 patients were classified as 2 (*n* = 3) and 3 (*n* = 4). When looking at PFS in relation to genotype, we found that there were no significant differences based on genotypes; however, those classified as “undetected” for genotype had significantly worse prognosis compared with all others (HR = 3.41, 95% CI: 1.66–6.99; *P* = 0.0008; Table 3).

**TABLE 3:** Cox proportional hazards model results for PFS and the influence of genotype classification. Separation reflects different models for different breakdowns of genotype group

Genotype group	*n*	HR (95% CI)	*P*-value
1a/1b/1	33	Ref	
2	3	0.84 (0.25–2.78)	0.77
3	4	2.40 (0.68–8.39)	0.17
Undetected	12	3.65 (1.72–7.73)	0.0007
1, 1a, 1b, 2, or 3	41	Ref	
Undetected	12	3.41 (1.66–6.99)	0.0008

## Discussion

This multigroup study of the addition of doxorubicin to sorafenib therapy did not show improvement of OS or PFS in patients with HCC. Although this study, like almost every other contemporary study in HCC, was restricted to patients who could have no worse than Child-Pugh grade A cirrhosis, HCC is a very heterogeneous disease, and similar patient populations can have very different outcomes (e.g., the SHARP North American/European and the Asia-Pacific sorafenib phase III trials; ref. 9).

Contrary to the preclinical hypothesis that sorafenib could efficiently block HCV replication and viral gene expression (2), we observed that sorafenib did not influence HCV titer levels in patients with HCC in this study. Only 2 patients showed conversion from detectable to undetectable HCV titer, one on each treatment arm. Patients treated with sorafenib, independent of the addition of doxorubicin, and who had HCV-UN had a poorer PFS compared with patients with higher titers of HCV titers at baseline; there was no difference in survival outcomes based on HCV genotype in patients with HCV infection. Our results are like the collaborative effort of two prior phase III clinical trials of sorafenib in Chinese patients with advanced HCC (12). It is important to note though that patients with HCV-UN had more T4 tumors thus more metastatic disease. Even if not statistically significant this may have influenced the suggested worse PFS. In all three studies, patients with HCV had better OS compared with patients with HBV infection (9, 10). The data presented herein, however, suggest that these outcomes are independent of any coincidental anti-HCC viral effect of the anticancer treatment.

Losses and gains of chromosome regions may differ in HCC caused by HBV as compared with HCV infection, which may partly explain the findings presented herein (10–13). The therapeutic field has evolved with the advent of checkpoint blockade as an effective treatment for HCC. The etiology of HCC is tightly connected to the activation of immune cells, in that CD8^+^ T cells help induce nonalcoholic steatohepatitis-HCC, rather than the anticipated immune surveillance (14). The impact of the tumor immune microenvironment extends beyond exhausted CD8^+^ T cells. An elevation in Ki67+ subset of CD8^+^ T cells leads to further improvement in outcome, independent of etiology, that is still more enhanced with the addition of anti-CTLA4 therapy (15).

Limitations of this correlative study include the potential invalidity of the hypothesis because novel data suggest a considerably more complex tumor immune microenvironment than originally thought, as solely impacted by the viral status etiology and extent (16, 17). In patients with chronic HCV, severe CD4^+^ and CD8^+^ T-cell dysfunctions have been reported. This suggests that HCV could employ certain mechanisms to counteract or suppress the host T-cell responses (18). An understanding of such implied molecular mechanisms has already started. Novel molecules that are carcinogenic to hepatitis viruses and activate the PI3K-Akt-mTOR pathway have been identified (19). These include five hub genes, POLR2A, POLR2B, RPL5, RPS6, and RPL23A, add to M2-type macrophages. These would be expected to serve as molecular markers and targets for diagnosis and treatment of HCC.

The small number of patients with HCV included and the small number of patient samples analyzed is also noted. When CALGB 80802 was initiated, tissue and biospecimen acquisition was optional out of concern that mandatory eligibility criteria might inhibit patient enrolment. Even though biospecimens were banked in only a subset of these patients, the correlative results provide meaningful data despite the disappointing clinical results.

Future investigation is needed on the outcomes of therapies based on the underlying etiology of HCC, particularly given the notable emergence of new therapies for HCC over the last half decade (20, 21).

To conclude, despite a strong preclinical rationale, we observed no difference in outcome for patients with HCC treated with sorafenib and observed an unexplained improvement in PFS in a small patient subset.

## Supplementary Material

Supplementary Figure 1 Supplementary Figure 1. CONSORT diagram of those with evaluable sample for the HCV-based analyses for this manuscript

Supplementary Figure 2 Supplementary Figure 2. Tile plots of HCV viral ჼ00ter levels over ჼ00me for paჼ00ents treated with Doxorubicin and Sorafenib (A) or Sorafenib alone (B). For sample collected at the progression ჼ00me point, the ჼ00ming of progression is reflected as text in that ჼ00le.

Supplementary Figure 3 Supplementary Figure 3. Kaplan-Meier curves for the progression-free survival distributions for patients who have detectable (HCV-D) vs. undetectable (HCV-UN) HCV titer levels on their first evaluated sample, by treatment arm.

Supplementary Figure 4 Supplementary Figure 4. Kaplan-Meier curves for the overall survival distributions for patients who have detectable (HCV-D) vs. undetectable (HCV-UN) HCV titer levels on their first evaluated sample, by treatment arm.
