# Supplementary material for: CALGB 80802 (Alliance): Impact of Sorafenib with and without Doxorubicin on Hepatitis C Infection in Patients with Advanced Hepatocellular Carcinoma
Source: Cancer Res Commun. 2024 Mar 7;4(3):682–90. doi: 10.1158/2767-9764.CRC-22-0516 (PMC10919207; doi:10.1158/2767-9764.CRC-22-0516)
Supplement: Supplementary Figure 1 — CONSORT diagram of those with evaluable sample for the HCV-based analyses for this manuscript [file crc-22-0516-s01.pdf]

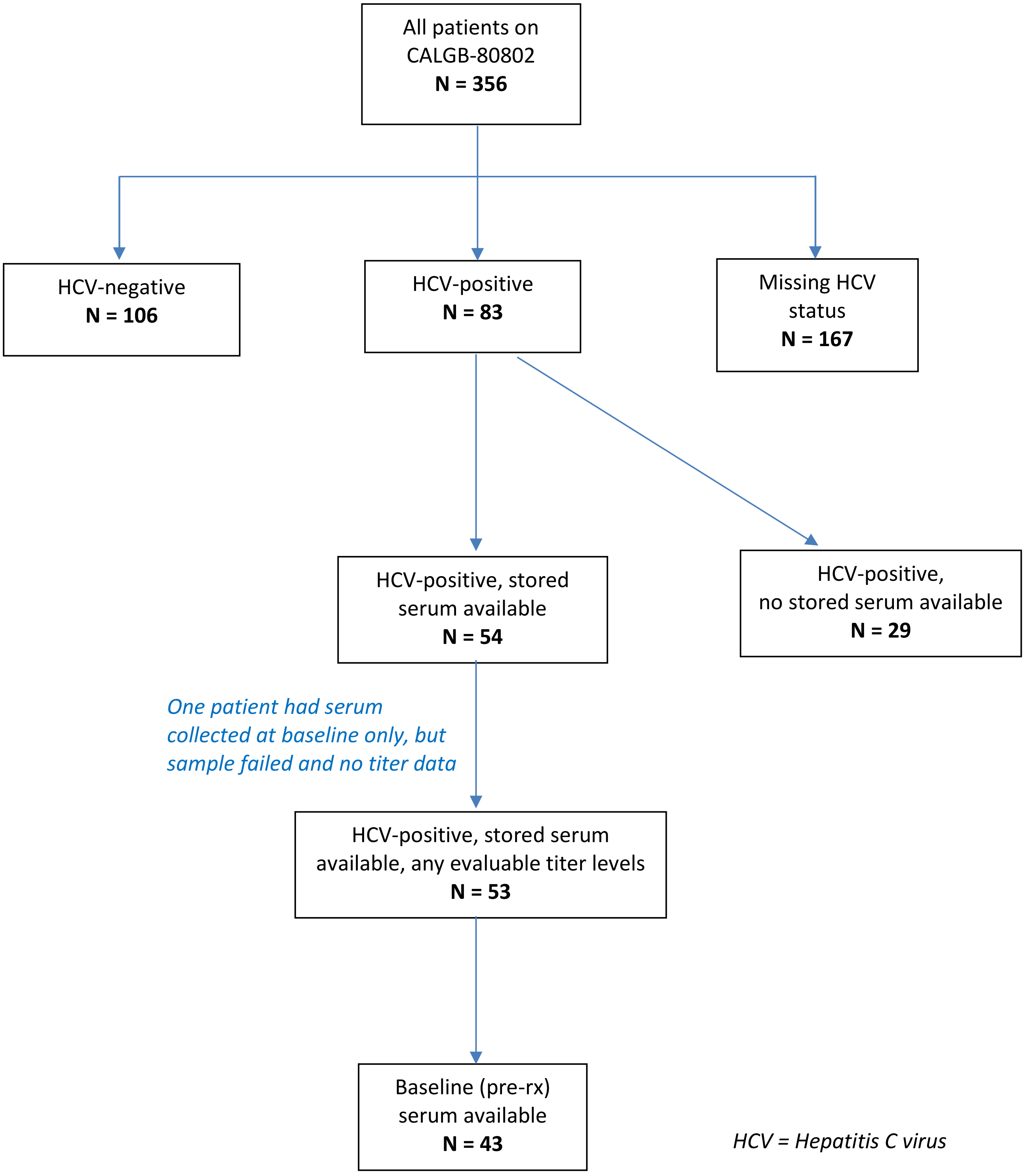

Supplemental Figure 1: CONSORT diagram of those with evaluable sample for the HCV-based analyses for this manuscript
