# Supplementary material for: CALGB 80802 (Alliance): Impact of Sorafenib with and without Doxorubicin on Hepatitis C Infection in Patients with Advanced Hepatocellular Carcinoma
Source: Cancer Res Commun. 2024 Mar 7;4(3):682–90. doi: 10.1158/2767-9764.CRC-22-0516 (PMC10919207; doi:10.1158/2767-9764.CRC-22-0516)
Supplement: Supplementary Figure 2 — Tile plots of HCV viral ჼ00ter levels over ჼ00me for paჼ00ents treated with Doxorubicin and Sorafenib (A) or Sorafenib alone (B). For sample collected at the progression ჼ00me point, the ჼ00ming of progression is reflected as text in that ჼ00le. [file crc-22-0516-s02.pdf]

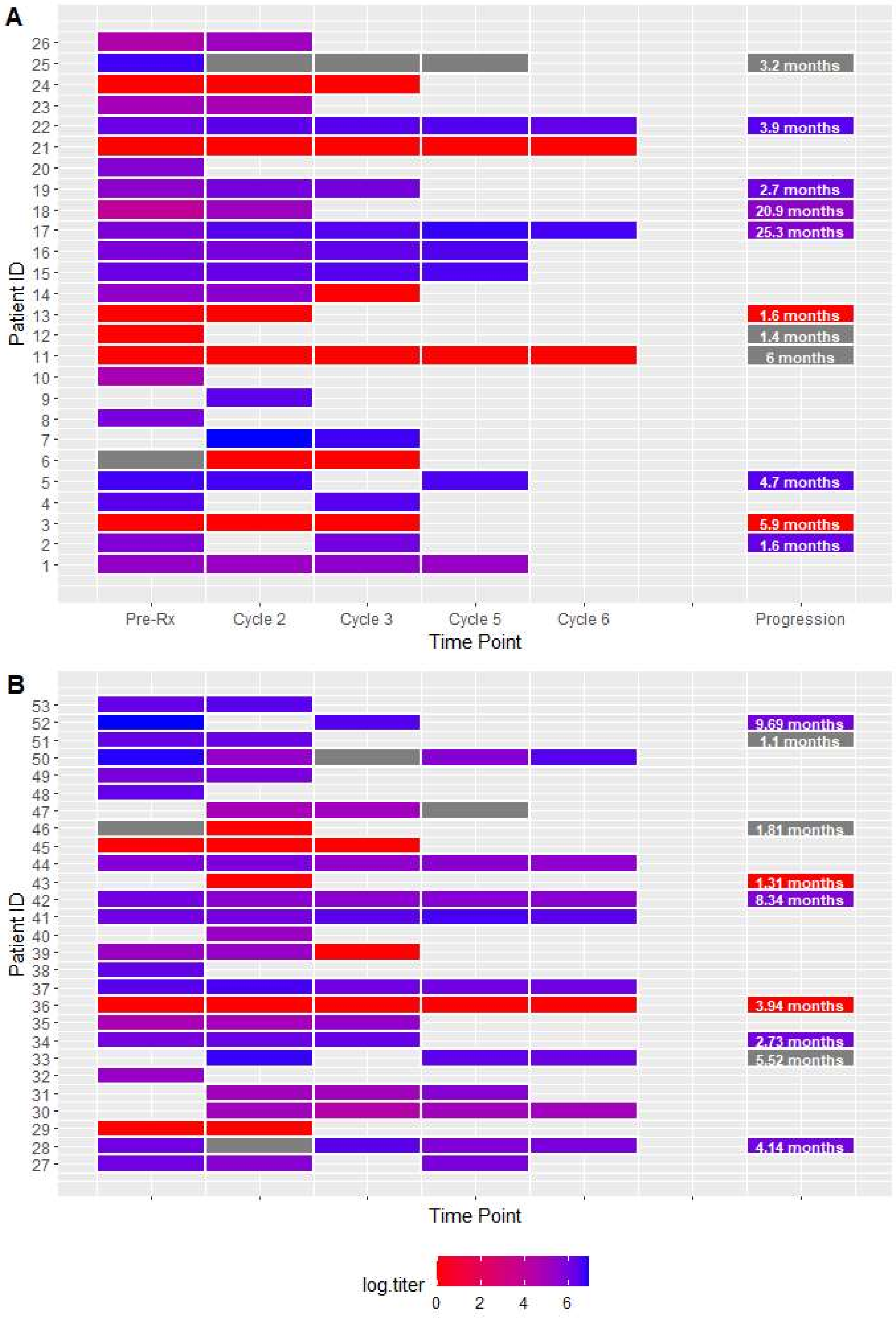

Supplemental Figure 2: Tile plots of HCV viral titer levels over time for patients treated with Doxorubicin and Sorafenib (A) or Sorafenib alone (B). For sample collected at the progression time point, the timing of progression is reflected as text in that tile.
