# Supplementary material for: CALGB 80802 (Alliance): Impact of Sorafenib with and without Doxorubicin on Hepatitis C Infection in Patients with Advanced Hepatocellular Carcinoma
Source: Cancer Res Commun. 2024 Mar 7;4(3):682–90. doi: 10.1158/2767-9764.CRC-22-0516 (PMC10919207; doi:10.1158/2767-9764.CRC-22-0516)
Supplement: Supplementary Figure 4 — Kaplan-Meier curves for the overall survival distributions for patients who have detectable (HCV-D) vs. undetectable (HCV-UN) HCV titer levels on their first evaluated sample, by treatment arm. [file crc-22-0516-s04.pdf]

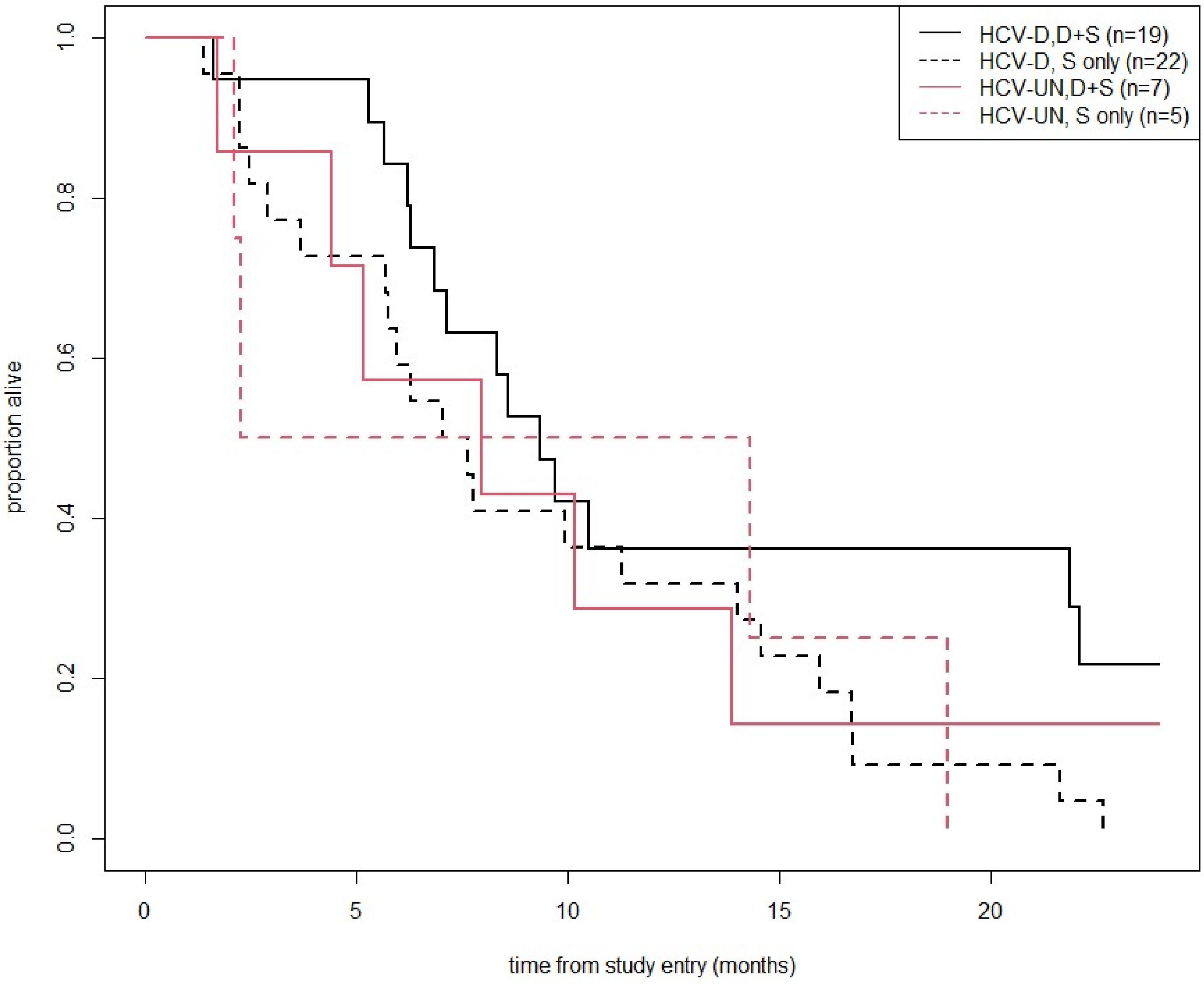

Supplemental Figure 4: Kaplan-Meier curves for the overall survival distributions for patients who have detectable (HCV-D) vs. undetectable (HCV-UN) HCV titer levels on their first evaluated sample, by treatment arm.
